# Molecular Imaging in Parathyroid Carcinoma Management: A Comprehensive Review

**DOI:** 10.3390/life15121861

**Published:** 2025-12-04

**Authors:** Petra Petranović Ovčariček, Luca Giovanella, Murat Tuncel, Junko Inoue Inukai, Virginia Liberini, Matija Romić, Désirée Deandreis, Rosaria Maddalena Ruggeri, Flaminia Vocaturo, Alfredo Campennì, Martin W. Huellner

**Affiliations:** 1 Department of Oncology and Nuclear Medicine, University Hospital Center Sestre Milosrdnice, 10000 Zagreb, Croatia; 2 School of Medicine, University of Zagreb, 10000 Zagreb, Croatia; 3 Department of Nuclear Medicine and Thyroid Center, Gruppo Ospedaliero Moncucco, Via Soldino 5, 6900 Lugano, Switzerland; 4 Department of Nuclear Medicine, University Hospital of Zurich, Rämistrasse 100, 8091 Zurich, Switzerland; 5 Department of Nuclear Medicine, University of Zurich, Pestalozzistrasse 3, 8032 Zurich, Switzerland; 6 Department of Nuclear Medicine, Hacettepe University, 06230 Ankara, Turkey; 7 Department of Radiology, Kobe University Graduate School of Medicine, 7-5-1, Kusunoki-cho, Chuo-ku, Kobe 650-0017, Hyogo, Japan; 8 Department of Nuclear Medicine, Azienda Ospedaliera S Croce e Carle Cuneo, Via Michele Coppino 26, 12100 Cuneo, Italy; 9 Institute Gustave Roussy, 94800 Villejuif, France; 10 Endocrinology Unit, Department of Human Pathology of Adulthood and Childhood DETEV, University of Messina, Via Consolare Valeria 1, 98125 Messina, Italy; 11 Department of Radiological Sciences and Haematology, Section of Nuclear Medicine, Università Cattolica del Sacro Cuore, 00168 Rome, Italy; 12 Unit of Nuclear Medicine, Department of Biomedical and Dental Sciences and Morpho-Functional Imaging, University of Messina, 98100 Messina, Italy

**Keywords:** parathyroid carcinoma, molecular imaging, [^18^F]fluorocholine, PET/CT, PET/MR, [^99m^Tc]Tc-MIBI, scintigraphy, ultrasonography, hyperparathyroidism, theranostics

## Abstract

Parathyroid carcinoma (PC) is an exceedingly rare endocrine malignancy, accounting for less than 1% of all primary hyperparathyroidism (pHPT) cases. It typically presents with pronounced hypercalcemia and markedly elevated parathyroid hormone (PTH) levels. Accurate imaging plays a pivotal role in diagnosis, staging, surgical planning, and long-term surveillance, although differentiating PC from benign disease on imaging remains a significant challenge. A multimodal imaging strategy combining cervical ultrasonography (US) and nuclear medicine techniques provides high sensitivity for lesion detection. Ultrasonography with advanced detective flow imaging allows detailed anatomical assessment and evaluation of vascular patterns of the primary tumor. [^99m^Tc]Tc-methoxyisobutylisonitrile ([^99m^Tc]Tc-MIBI) scintigraphy frequently demonstrates prolonged tracer retention in PC, while [^18^F]fluorocholine positron emission tomography/computed tomography (PET/CT) and positron emission tomography/magnetic resonance (PET/MR) imaging have shown superior performance for detecting both primary tumors and metastatic disease due to its higher spatial resolution and higher molecular sensitivity. [^18^F]FDG PET serves as an adjunct modality for identifying aggressive, metabolically active lesions. Emerging radiotracers such as [^18^F]-fibroblast activation protein inhibitor ([^18^F]FAPI) and [^68^Ga]Ga-trivehexin have shown potential in cases where initial imaging is inconclusive. Theranostic strategies that integrate molecular imaging with targeted radioligand therapy may transform PC management by enabling personalized treatment approaches tailored to each tumor’s biological and imaging characteristics. This review aims to evaluate available imaging modalities for PC diagnosis and provide guidance for their clinical application.

## 1. Introduction

Parathyroid carcinoma is an exceptionally rare endocrine malignancy, with an estimated incidence of 3.5 to 5.7 cases per 10 million individuals. The disease was first described in 1904 by Swiss surgeon Fritz de Quervain, who referred to it as “parastruma maligna aberrata” [1,2]. Globally, the disease represents less than 0.005% of all cancers [3] and accounts for fewer than 1% of pHPT cases [4]. The median age at diagnosis is 62 years, with a slightly higher prevalence among men, particularly within Caucasian populations (around 75% of cases) [5]. Clinically, PC is characterized by markedly elevated PTH levels and significant hypercalcemia, leading to a spectrum of symptoms that range from mild fatigue and nausea to severe complications such as renal failure and pathological fractures [6,7,8,9]. Although PC generally follows a slow growth pattern, it carries substantial morbidity due to recurrent or persistent hypercalcemia and its tendency to metastasize, most commonly to the bones, lungs, and liver [8].

The most common clinical manifestations of PC arise from skeletal and renal complications secondary to severe hyperparathyroidism. Bone-related symptoms include pain, pathological fractures, and osteoporosis, while renal manifestations such as nephrolithiasis and renal insufficiency are also frequent. Less common but clinically relevant findings include generalized weakness (asthenia) and cervical compression symptoms—such as dysphonia, dysphagia, or dyspnea—resulting from tumor mass effect, which is palpable in approximately 15–50% of cases. Additional signs of severe hypercalcemia may include polyuria–polydipsia syndrome, nausea, vomiting, neuropsychiatric disturbances, peptic ulcer disease, and acute pancreatitis [10,11].

From a biochemical standpoint, hypercalcemia in PC is typically severe, with serum calcium often exceeding 140 mg/L (3.5 mmol/L). Elevated alkaline phosphatase and markedly increased PTH levels (ranging from three- to tenfold above the upper reference limit) are characteristic findings [4]. In a retrospective study of 131 Japanese patients with pHPT (111 with benign parathyroid lesions and 20 with PC), those with PC exhibited significantly higher mean serum calcium (12.7 mg/dL vs. 11.6 mg/dL) and PTH levels (397 pg/mL vs. 228 pg/mL) compared with benign cases [12]. Circulating PTH thus remains a key biomarker for detecting recurrence and monitoring disease progression.

PC may occasionally present with acute symptoms, including encephalopathy, vomiting, cardiac arrhythmia, and acute renal failure, all associated with a significant rise in PTH levels and severe hypercalcemia, the so-called parathyroid crisis. This represents a medical emergency that requires rapid intervention to manage hypercalcemia, typically through the administration of intravenous isotonic saline, with or without loop diuretics. Additional treatments, such as biphosphonates, cinacalcet, and calcitonin, can provide temporary relief. However, the definitive treatment of parathyroid crisis remains surgical parathyroidectomy [13].

The main goals of surgical management in PC are to evaluate tumor invasiveness, prevent further metastatic dissemination, and alleviate the systemic effects of excessive PTH secretion [14]. Achieving these objectives requires a precise and thorough preoperative diagnostic assessment to guide the optimal treatment strategy. The surgical state-of-the-art treatment for localized PC is an en bloc resection of the parathyroid tumor along with surrounding adipose tissue, the ipsilateral thyroid lobe, combined with ipsilateral central neck dissection to ensure a continuous margin of healthy tissue [14].

In most cases, PC is not recognized before surgery; the diagnosis is usually confirmed by histopathology following resection. The disease is characterized by its aggressive clinical behaviour, with regional lymph node metastases observed in 15–30% and distant metastasis in up to 30% at the time of diagnosis. Consequently, effective treatment often requires a multimodal approach integrating surgery with adjuvant or targeted therapies to address both local and systemic disease components.

Patients with metastatic PC generally have poor prognoses, especially when metastases are hormonally active and continue to secrete PTH. Surgical intervention remains the cornerstone of management, as both external radiotherapy and chemotherapy have shown limited therapeutic benefit. Reported five-year overall survival ranges from 60% to 95%, while recurrence occurs in approximately 30–67% of cases. These figures highlight the critical importance of advanced imaging techniques for early diagnosis, precise staging, and vigilant long-term monitoring [4].

Imaging plays an increasingly vital role in PC management—facilitating accurate tumor localization, differentiation from benign lesions where feasible, assessment of local invasion and distant metastases, surgical planning, and detection of recurrence. This comprehensive review explores the full spectrum of diagnostic imaging strategies for PC, emphasizing recent technological advances in diagnostic and monitoring capabilities. Modern approaches integrate multiple complementary modalities, including US with detective flow imaging and advanced nuclear medicine techniques, the latter offering not only superior diagnostic accuracy but also promising avenues for future therapeutic applications.

This review aims to comprehensively evaluate available imaging modalities for PC diagnosis, compare their diagnostic accuracy and clinical utility, and provide practical guidance for imaging strategy selection in suspected PC cases.

## 2. Ultrasonography

Ultrasonography is a key diagnostic modality for detecting parathyroid lesions and is widely used as the first-line imaging technique owing to its broad availability, non-invasiveness, cost-effectiveness, and ability to provide real-time evaluation of cervical anatomy. It plays a central role in the initial assessment of suspected parathyroid disease and can offer valuable clues suggestive of malignancy.

**Figure 1:**
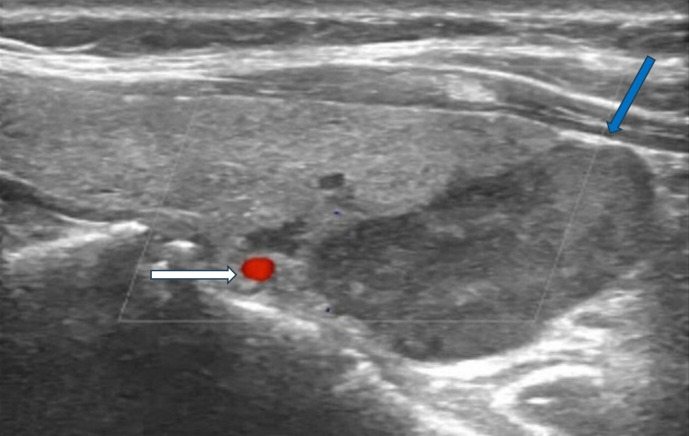
Ultrasonography image of parathyroid carcinoma. **Legend:** 65-year-old patient with parathyroid carcinoma presented with serum PTH of 1300 pg/mL (12–88 pg/mL) and Ca:13 mg/dL (8.8–10.6 mg/dL). Ultrasonography showed a hypoechoic mass with lobulated borders (32 × 23 × 14 mm) next to the lower pole of the left thyroid lobe (blue arrow). The white arrow shows the inferior thyroid artery.

Sonographic features indicative of PC typically include lesion size greater than 3 cm, substantially larger than typical benign adenomas, which generally measure 1–2 cm. Malignant lesions often exhibit irregular or lobulated margins with evidence of invasion into adjacent tissues, in contrast to the smooth, well-circumscribed borders characteristic of benign counterparts (Figure 1).

**Figure 2:**
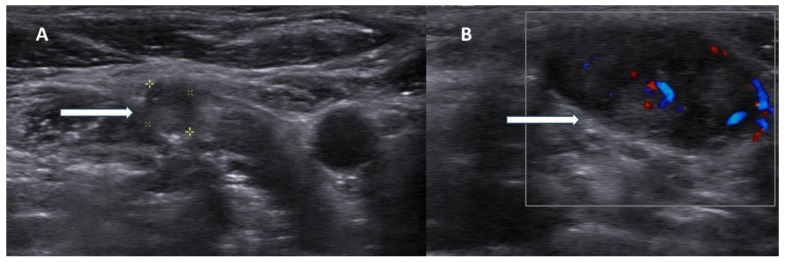
Ultrasonography images of parathyroid carcinoma neck metastases. **Legend**: 70-year-old patient presented with parathyroid carcinoma lymph node metastases with hypoechoic lesions without fatty hilum ((**A**), white arrow) and chaotic blood flow in Doppler imaging ((**B**), white arrow).

Additional suspicious findings include a heterogeneous echotexture with marked hypoechogenicity, reflecting necrosis, hemorrhage, or fibrosis, and a depth-to-width ratio of ≥1, as malignant lesions tend to be taller than wide. The presence of intralesional calcifications and cervical lymphadenopathy further strengthens the suspicion for carcinoma [2,15,16] (Figure 2).

However, these sonographic characteristics often overlap with those of large or atypical benign adenomas, and no single US feature is pathognomonic for malignancy. Conventional US achieves a sensitivity of approximately 76.1% and a positive predictive value (PPV) of approximately 93.2% for identifying parathyroid lesions [17], yet its ability to distinguish benign from malignant pathology remains limited. Moreover, as an operator-dependent technique, US demands substantial experience and specialized expertise in parathyroid imaging to ensure accuracy and reproducibility (Table 1).

Recent technological advances have improved the diagnostic performance of US through detective flow imaging, a novel technique that surpasses traditional color Doppler in detecting slow microvascular blood flow. A 2024 study by Matsui et al. highlights its clinical utility, demonstrating successful visualization of low-velocity flow patterns within parathyroid tumors. This method enabled the detection of vascular-rich PC and provided valuable insights into lesion microvascularity, thereby enhancing diagnostic confidence in differentiating PC from benign disease [18].

While detective flow imaging offers a promising non-invasive advancement in enhancing the diagnostic accuracy of US, several important limitations must be acknowledged. The method requires dedicated high-performance equipment and operators with specialized training, which may restrict its availability to expert centers. Furthermore, larger prospective validation studies are needed to define standardized diagnostic criteria, establish sensitivity and specificity for malignancy detection, and ensure reproducibility across institutions.

The use of US-guided fine needle aspiration cytology (FNAC) for parathyroid lesions is a matter of discussion. The procedure can be performed with several passes to obtain enough cells for cytological evaluation or it can be performed as a simple PTH washout test, in which the physician performs fewer passes and rinses the biopsy needle with a saline solution to detect PTH levels; i.e., fine-needle aspiration-PTH (FNA-PTH). FNA-PTH washout test is a safe procedure with low risk of complications and high sensitivity and specificity (≥90%) for parathyroid lesions [19,20,21]. From a procedural standpoint, although no data for only FNA-PTH is available, FNAC is contraindicated in suspected PC for two key reasons: first, cytology cannot reliably differentiate benign from malignant parathyroid lesions due to overlapping cellular morphology; and second, there is a risk of tumor seeding along the needle tract, potentially leading to local recurrence or dissemination of malignant cells [22,23,24]. However, the decision for FNA may be challenging. If there is a high suspicion of PC or if there is a recurrent lesion after parathyroid cancer surgery, FNAC should be avoided. In such cases, the necessity for FNAC is also questionable, since surgical excision is recommended.

In parallel, recent advances in artificial intelligence have introduced new opportunities for non-invasive diagnosis. A recent retrospective study involving 913 surgically confirmed cases (823 benign adenomas, 90 malignant or atypical lesions) applied radiomics-based machine learning models to US imaging [25]. Among the tested algorithms, the Random Forest model demonstrated the highest diagnostic performance (AUC 0.933, accuracy 0.940), significantly outperforming support vector machine and logistic regression classifiers. These findings highlight the potential of radiomics-assisted US as a valuable preoperative tool for distinguishing PC from benign adenomas and for improving clinical decision-making in parathyroid imaging.

## 3. [^99m^Tc]Tc-MIBI Scintigraphy

[^99m^Tc]Tc-MIBI scintigraphy of the parathyroid glands, first described by Coakley et al. in 1989 [26], remains a valuable imaging technique for the evaluation of PC, as these tumors generally exhibit intense radiotracer uptake. [^99m^Tc]Tc-MIBI is a lipophilic, cationic compound widely used in parathyroid imaging that accumulates within mitochondria-rich cells, thereby reflecting both perfusion and metabolic activity. Uptake occurs in two phases: an initial distribution phase driven by tissue perfusion and cellular metabolism, followed by mitochondrial sequestration linked to the high negative transmembrane potential. Differential tracer washout between parathyroid and thyroid tissue forms the basis of lesion detection [27,28,29,30].

Standard dual-phase protocols involve early-phase imaging at 10–15 min post-injection to assess initial distribution and delayed-phase imaging at 1.5–2.5 h post-injection to assess tracer retention. In suspected PC, whole-body imaging is recommended to assess for potential metastatic spread. Single-photon emission computed tomography/computed tomography (SPECT/CT) enhances planar imaging by providing three-dimensional localization and improved anatomical correlation. While normal parathyroid and thyroid tissues exhibit rapid [^99m^Tc]Tc-MIBI washout, hyperfunctioning parathyroid tissue –both benign and malignant—demonstrates prolonged retention, allowing for distinction on delayed imaging. Alternatively, subtraction scintigraphy combining [^99m^Tc]Tc-MIBI with thyroid tracers such as [^99m^Tc]pertechnetate or Na [^123^I]I, improves the detection and localization of hyperfunctioning parathyroid lesions beyond that achieved with dual-phase protocols [31,32,33,34].

**Figure 3:**
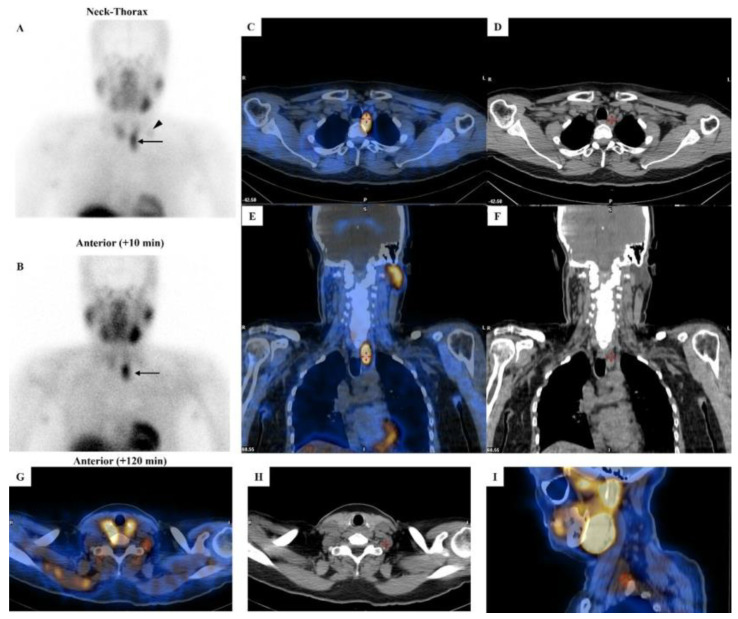
[^99m^Tc]Tc-MIBI imaging in a patient with parathyroid carcinoma. **Legend**: A 52-year-old man affected by pHPT [intact PTH = 421.6 pg/mL (8–76), total calcium = 12.1 mg/dL (8.2–10.4), ionized calcium = 1.82 mmol/L (1.10–1.30)] due to PC. Neck ultrasound showed a hypoechoic and heterogeneous parathyroid lesion (28 mm in maximum size) close to the lower pole of the left thyroid lobe. Dual-phase parathyroid scintigraphy was performed using [^99m^Tc]Tc-MIBI. Panels (**A**,**B**): Planar parathyroid images (anterior views) were obtained 10 and 120 min after [^99m^Tc]Tc-MIBI administration (400 MBq). Early (panel (**A**)) and late (panel (**B**)) images showed a well-defined area of abnormal and intense tracer uptake located at the lower pole of the left thyroid lobe (black arrow). At visual assessment (i.e., qualitative analysis), the late image (panel (**B**)) showed a significant tracer retention in the parathyroid lesion. Using a semiquantitative approach, the peak Retention Index (RI) was suspicious for PC (peak RI = 11.45%). Panels (**C**,**E**): SPECT/CT imaging was acquired 130 min after [^99m^Tc]Tc-MIBI administration. Axial (panel (**C**)) and coronal (panel (**E**)) images confirmed an abnormal tracer uptake located lower than the inferior pole of the left thyroid lobe (red cross-mark). At neck-thorax CT performed without contrast agent media administration (panels (**D**,**F**)), a large-sized (28 mm in maximum diameter) and non-homogeneous parathyroid lesion was noted just lower with respect to the inferior pole of the left thyroid lobe (red cross-mark). In addition, a faint but focal and abnormal tracer uptake was noted at the early planar image (panel (**A**)) in the middle-lower part of the left lateral neck (black arrowhead), consistent with lymph-node metastasis, which was then confirmed by hybrid imaging (red cross-mark in panels (**G**–**I**)).

When combined with the US, [^99m^Tc]Tc-MIBI scintigraphy achieves sensitivities of 81–95% for detecting hyperfunctioning parathyroid lesions [35,36,37]. Importantly, tracer retention patterns may suggest malignancy. Zhang et al. demonstrated that PCs exhibit significantly higher retention indices compared to benign parathyroid lesions, with distinct differences in both mean and peak retention values [38]. This finding highlights the potential of retention analysis as a non-invasive preoperative tool for differentiating malignant from benign parathyroid disease (Figure 3).

Semi-quantitative evaluation methods—such as delayed-to-early uptake ratio, tumor-to-background ratio on delayed images, and washout rate calculations—have been explored for preoperative differentiation [39]. However, retention indices can overlap between benign and malignant lesions, particularly in small tumors. Hence, further validation and methodological standardization are needed before these parameters can be integrated into routine clinical practice.

Interestingly, some reports describe absent tracer uptake in the primary tumor but increased uptake in metastases, underscoring the heterogeneous tracer kinetics of PC [40]. Despite the valuable diagnostic insights provided by [^99m^Tc]Tc-MIBI scintigraphy, complementary imaging with [^18^F]fluorocholine PET/CT(MR) imaging is often required, given its superior sensitivity and spatial resolution for detecting both primary and metastatic disease [2] (Table 2).

## 4. [^18^F]fluorocholine PET

PET/CT(MR) imaging has emerged as a powerful tool for evaluating PC, offering superior spatial resolution and sensitivity compared to [^99m^Tc]Tc-MIBI scintigraphy. Imaging with ^18^F-labeled choline analogs demonstrates high diagnostic efficacy for detecting both primary and metastatic PC lesions, with growing evidence supporting its value [2], even in non-secreting PTH cases [41].

**Figure 4:**
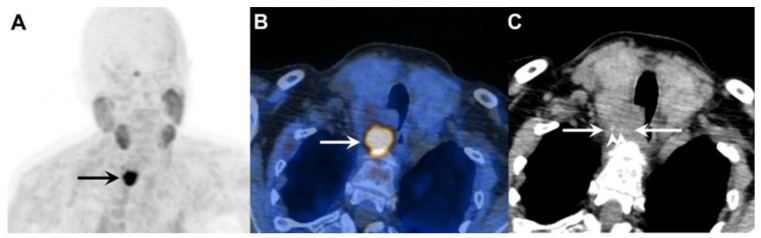
A 73-year-old woman with primary hyperparathyroidism due to parathyroid carcinoma. **Legend**: (**A**) Coronal [^18^F]fluorocholine PET maximum intensity projection (MIP) image demonstrates an intensely tracer-avid lesion in the neck (black arrow, SUV_max_ 18.9). (**B**) Axial fused [^18^F]fluorocholine PET/CT image shows the tumor (white arrow) located deep to the right lobe of the enlarged thyroid gland. (**C**) On the corresponding unenhanced CT image, there is loss of the fat plane between the tumor (white arrows) and surrounding structures, with intralesional stippled calcifications visible (white arrowheads).

[^18^F]fluorocholine is a PET tracer that undergoes phosphorylation by choline kinase, an enzyme central to phospholipid synthesis in cell membranes. Hyperfunctioning parathyroid tissue—particularly adenomas and carcinomas—shows upregulated choline metabolism, leading to marked tracer accumulation. Compared to traditional scintigraphy, [^18^F]fluorocholine PET/CT(MR) imaging provides several key advantages: markedly improved spatial resolution (4–5 mm vs. 10–15 mm), shorter acquisition time (approximately 30 min vs. 2–3 h), higher contrast-to-background ratio, and lower radiation exposure, particularly when performed with PET/MR imaging [42]. Figure 4. demonstrates an example of PC detected on [^18^F]fluorocholine PET/CT.

Several case reports and small cohort studies have confirmed the ability of [^18^F]fluorocholine PET to detect primary PC, local recurrences, regional lymph node metastases, and distant metastases to sites such as bone and lung, as well as other organs [41,43,44,45]. Notably, one case demonstrated the value of this modality in identifying an [^18^F]fluorocholine-positive PC metastasis in the skull base, which was subsequently removed via a transnasal-transpterygoid endoscopic approach [46]. Another report described bone and pulmonary lesions visible on [^18^F]fluorocholine PET but not on [^18^F]FDG PET/CT, highlighting the complementary role of both tracers [47].

A notable limitation, as with all radiotracers, is that [^18^F]fluorocholine PET cannot reliably distinguish benign adenomas from localized PC, as both demonstrate increased choline metabolism with overlapping standardized uptake values (SUV). However, the presence of a large cervical mass with intralesional calcifications, in conjunction with pathologic lymph nodes or lesions suspicious for distant metastases, is highly suggestive of PC rather than benign disease. Hence, when malignancy is clinically suspected, whole-body [^18^F]fluorocholine PET/CT(MR) imaging is recommended for comprehensive staging.

## 5. [^11^C]-Labeled Tracers

Carbon-11-labeled choline ([^11^C]CH) tracers share a similar uptake mechanism with [^18^F]fluorocholine and can be useful, particularly when conventional imaging methods such as US and [^99m^Tc]Tc-MIBI scintigraphy fail to localize parathyroid lesions [48,49,50]. However, evidence supporting their effectiveness in detecting PC is lacking.

[^11^C]methionine ([^11^C]MET) has been applied as a second-line imaging option for identifying hyperfunctioning parathyroid tissue in cases with inconclusive conventional imaging, demonstrating higher sensitivity than [^99m^Tc]Tc-MIBI or US [51,52,53,54]. Nonetheless, its performance is inferior to that of [^18^F]fluorocholine [55,56,57] and [^11^C]CH [57]. [^11^C]MET uptake reflects amino acid metabolism and PTH precursor synthesis, providing complementary metabolic information distinct from [^18^F]FDG or [^18^F]fluorocholine [30,52,58]. In an isolated report, [^11^C]MET PET has successfully detected local recurrence even when both [^99m^Tc]Tc-MIBI and [^18^F]FDG PET/CT were negative [59].

The major limitation of carbon-11-labeled tracers is their short physical half-life of approximately 20 min, necessitating on-site cyclotron production. This logistical constraint restricts their availability to specialized centers and limits their broader clinical use in PC imaging compared to [^18^F]fluorocholine. Another limitation is their inferior imaging characteristics.

## 6. ^18^F-Fluorodeoxyglucose PET

^18^F-fluorodeoxyglucose ([^18^F]FDG) serves as a complementary imaging modality in PC assessment, particularly for evaluating treatment response in advanced disease. As a glucose analog, [^18^F]FDG accumulates in tissues with high glycolytic activity [60,61]. Benign parathyroid lesions generally demonstrate low (to moderate) [^18^F]FDG uptake (with a variable sensitivity of 0–94% and a PPV of 62–100% [62]), whereas PCs frequently exhibit markedly increased [^18^F]FDG avidity, consistent with their aggressive biological behavior and higher proliferation rate [47,63,64,65,66,67]. [^18^F]FDG PET/CT (MR) imaging is especially valuable for detecting regional and distant metastases of less differentiated PCs that exhibit increased glucose consumption [16].

Key indications for [^18^F]FDG PET/CT (MR) imaging in PC encompass initial staging when PC is confirmed or highly suspected to assess for local invasiveness as well as distant metastases. Aggressive disease is often characterized by substantially elevated calcium and PTH levels reflecting their biology. Other important indications include surveillance monitoring of metastatic disease and treatment response assessment [16,63]. In cases with aggressive local tumor progression or metastatic disease, [^18^F]FDG PET may complement [^18^F]fluorocholine by localizing lesions with high metabolic activity [2,67].

A critical interpretive limitation lies in the potential misidentification of brown tumors or osteolytic lesions secondary to severe pHPT as metastases [68,69,70,71,72]. Brown tumors—benign osteolytic lesions driven by excess osteoclast activity—often display moderate, sometimes high metabolic activity on [^18^F]FDG PET, presumably owing to the elevated metabolic activity of osteoclasts and accompanying inflammatory cells [73,74]. Correlation with CT or MR imaging is therefore essential, as brown tumors typically appear as well-circumscribed lytic “soap-bubble” lesions, which are commonly found in the mandible, clavicle, ribs, and pelvis [75]. Resolution of uptake following correction of hypercalcemia further supports their benign nature, while biopsy may be reserved for ambiguous cases, given the risk of tumor seeding.

## 7. Dual-Tracer PET Strategy

A sequential dual-tracer PET strategy combining [^18^F]fluorocholine and [^18^F]FDG offers complementary insights into PC biology and may provide substantially enhanced disease staging and progression monitoring. Whereas [^18^F]fluorocholine reflects increased cell membrane turnover via upregulated specific choline metabolism, [^18^F]FDG highlights glycolytic activity associated with tumor aggressiveness. Utilizing both tracers would allow for the detection of lesions with distinct metabolic phenotypes—ranging from highly proliferative, glucose-avid tumors to those exhibiting lower glycolytic rates but active membrane synthesis.

Clinical evidence supports the diagnostic synergy of this approach. Several reports have shown [^18^F]fluorocholine PET/CT identifying additional metastatic foci not visualized with [^18^F]FDG PET/CT alone, thereby broadening the scope of metastatic disease evaluation [43,47,67]. Conversely, more aggressive or dedifferentiated lesions may demonstrate preferential [^18^F]FDG uptake.

Accordingly, the dual-tracer strategy holds particular value for initial staging in confirmed PC, comprehensive preoperative assessment prior to resection of metastases, and selection of optimal biopsy sites, providing a more complete depiction of tumor heterogeneity and metabolic activity.

## 8. Novel Tracers and Potential Applications

Recent advances in molecular imaging have introduced novel PET tracers that may expand diagnostic and therapeutic possibilities in parathyroid carcinoma. A recent case report suggests that [^18^F]FAPI PET/CT can effectively detect PC metastases, particularly in patients with biochemical evidence of disease recurrence but negative findings in [^18^F]FDG and choline-based imaging [76]. Fibroblast Activation Protein Inhibitor tracers selectively target cancer-associated fibroblasts within the tumor microenvironment. This observation highlights the potential role of [^18^F]FAPI not only in improved disease localization but also in the emerging field of receptor-directed theranostics, potentially enabling personalized radioligand therapy in PC management.

Similarly, another novel radiotracer, [^68^Ga]Ga-Trivehexin, targets the αvβ_6_ integrin receptor and has shown promise in early clinical application [77]. In a recently reported case of recurrent PC, [^68^Ga]Ga-Trivehexin PET/CT successfully detected both local recurrence and pulmonary metastases, whereas [^18^F]FDG PET/CT and [^99m^Tc]Tc-MIBI SPECT/CT yielded negative or inconclusive findings. These preliminary results suggest that integrin-targeted imaging may be valuable when conventional modalities fail, warranting further systematic investigation.

**Figure 5:**
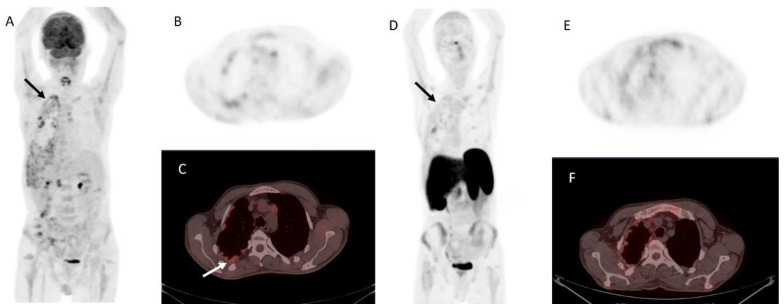
Metastatic parathyroid carcinoma on [^18^F]FDG PET/CT and [^68^Ga]Ga DOTA-TATE PET/CT. **Legend**: A 72-year-old patient with metastatic parathyroid carcinoma. He had elevated Ca levels of 13.5 mg/dL (8.8–10.6 mg/dL) and PTH levels of 11,663 (12–88 pg/mL) after repeated surgeries and tyrosine kinase inhibitor therapy. [^18^F]FDG PET/CT shows extensive pleural metastases ((**A**), whole-body MIP image, black arrow; (**B**), axial MIP; (**C**), PET/CT, white arrow). [^68^Ga]Ga DOTA-TATE PET/CT was acquired to evaluate options for peptide receptor radionuclide therapy ((**D**), whole-body MIP image; (**E**), axial MIP; (**F**), PET/CT). Unfortunately, there was a faint uptake lower than the physiological liver uptake, rendering the patient ineligible for PRRT.

In addition, parathyroid tissue—both benign and malignant—may express somatostatin receptors (SSTR) [78]. A study by Storvall et al. demonstrated SSTR expression across all parathyroid tumor subtypes—including typical adenomas, atypical adenomas, and PCs—predominantly within the cytoplasm rather than on the cell membrane. Among receptor subtypes, SSTR 1 showed uniform expression, while SSTR 2–5 varied by tumor type. Notably, PC exhibited consistently high levels of SSTR expression, particularly SSTR 5, while adenomas demonstrated only minimal expression. The marked difference in cytoplasmic SSTR 5 expression rates between parathyroid tumors may serve as a potential malignancy indicator [79]. These findings support the biological rationale for SSTR-based imaging [80] and potential theranostic applications using peptide receptor radionuclide therapy (PRRT) (Figure 5).

**Figure 6:**
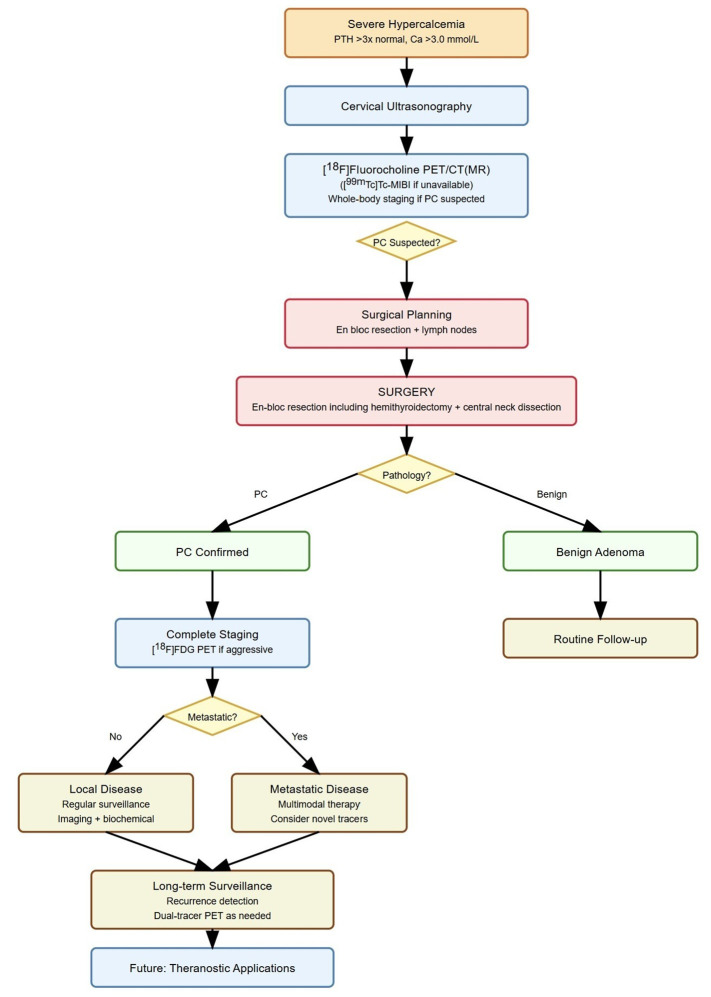
Molecular imaging in parathyroid carcinoma management. **Legend**: PC, parathyroid carcinoma; PET/CT(MR), positron emission tomography/computed tomography (magnetic resonance) imaging.

However, clinical evidence remains limited. At present, SSTR imaging is not recommended as a standard diagnostic tool in PC but may be considered selectively in patients with high receptor expression where PRRT is being contemplated as a therapeutic option (Figure 6).

Although no direct head-to-head comparison of [^99m^Tc]Tc-MIBI and [^18^F]fluorocholine in PC has been published, the available data on diagnostic accuracy and management impact clearly favor [^18^F]fluorocholine. Additionally, whole-body cross-sectional imaging with [^18^F]fluorocholine is much quicker than with [^99m^Tc]Tc-MIBI, taking approximately 15 min for [^18^F]fluorocholine PET/CT or PET/MR imaging, compared to about 2 h for [^99m^Tc]Tc-MIBI SPECT/CT.

## 9. Conclusions

The diagnosis and management of PC have evolved substantially with the integration of US and molecular imaging technologies. Ultrasonography, particularly when enhanced by detective flow imaging, has improved the assessment of tumor vascularity and tissue architecture, although its accuracy remains highly operator-dependent. Nuclear medicine imaging continues to play a pivotal role in longitudinal monitoring. In selected cases, combining nuclear medicine imaging techniques—such as dual-tracer PET imaging with [^18^F]fluorocholine and [^18^F]FDG—can provide complementary biological information, supporting more precise staging and targeted treatment planning. Whole-body imaging is essential to identify distant metastases, distinguishing PC from benign parathyroid lesions, where imaging is typically confined to the neck and mediastinum.

Emerging novel PET tracers such as [^18^F]FAPI and [^68^Ga]Ga-trivehexin have demonstrated potential in identifying PC lesions that are undetectable with other tracers, offering new diagnostic pathways in complex or recurrent cases.

Looking ahead, the future of PC imaging is likely to be shaped by the development of theranostic strategies that integrate diagnostic imaging with targeted radioligand therapy. Such precision medicine approaches, grounded in the molecular and metabolic characteristics of individual tumors, could transform PC management from a predominantly surgical disease to one guided by personalized, image-based systemic treatment paradigms.

## Figures and Tables

**Table 1:** Sonographic features suggestive of parathyroid carcinoma.

Feature	Malignant Characteristics	Benign Characteristics
**Size**	>3 cm	Typically <2 cm
**Margins**	Irregular, lobulated, thick capsule	Smooth, well-circumscribed
**Echogenicity**	Marked hypoechogenicity, heterogeneous	Homogeneous
**Shape**	Depth-to-width ratio ≥1 (taller than wide)	Width > depth
**Calcifications**	Intralesional calcifications present	Less common
**Lymph nodes**	Cervical lymphadenopathy	Absent
**Invasion**	Evidence of adjacent tissue invasion	Well-defined borders
**Vascularity**	Intralesional disordered hypervascularity	Polar artery

**Table 2:** Imaging modalities for parathyroid carcinoma.

Modality	Mechanism	Advantages	Limitations	Clinical Role
**Ultrasonography**	High-frequency sound waves	Non-invasive, real-time, cost-effective, widely available	Operator-dependent, specificity is low (cancer/adenoma)	First-line Complementary to [^99m^Tc]Tc-MIBI scintigraphy/[^18^F]FCH PET
**[^99m^Tc]Tc-MIBI scintigraphy**	Mitochondrial accumulation, lipophilic cationic compound	Functional information, whole-body imaging	Lower resolution, longer acquisition time, limited in differentiating benign from malignant parathyroid lesions	If [^18^F]FCH PET is unavailable
**[^18^F]Fluorocholine ([^18^F]FCH) PET**	Choline kinase phosphorylation, membrane synthesis	High resolution (4–5 mm), short acquisition time (~30 min), whole-body imaging, monitoring	Cannot distinguish between benign and malignant primary lesion	1st-line PET tracer
**[^18^F]FDG PET**	Glucose metabolism	High resolution (4–5 mm), short acquisition time (~30 min), detects aggressive lesions, whole-body imaging	Low sensitivity for differentiated PC forms	Complementary to [^18^F]FCH PET
**[^11^C]Choline PET**	Choline kinase phosphorylation, membrane synthesis	High-resolution (4–5 mm), short acquisition time (~30 min), whole-body imaging	Short half-life, on-site production needed	2nd-line PET tracer
**[^11^C]Methionine PET**	Amino-acid metabolism, PTH synthesis	Alternative when other tracers fail	Short half-life, on-site production needed	2nd-line PET tracer
**[^18^F]FAPI PET**	Cancer-associated fibroblasts	Theranostic potential	Limited evidence	Experimental
**[^68^Ga]** **Ga-Trivehexin PET**	αvβ_6_integrin receptor	Theranostic potential	Limited evidence	Experimental
**SSTR PET tracers**	Somatostatin receptors	Theranostic potential	Limited evidence	Experimental

**Legend**: FAPI, Fibroblast Activation Protein Inhibitor; PET, positron emission tomography; SSTR, somatostatin receptors.

## Data Availability

No new data were created or analyzed in this study.
